# Case report : a novel *ASXL3* gene variant in a Sudanese boy

**DOI:** 10.1186/s12887-021-03038-8

**Published:** 2021-12-09

**Authors:** Ke Wu, Yan Cong

**Affiliations:** 1 Prenatal Diganosis Center, Yiwu Maternity and Child Health Care Hospital, Xinke Road C100, Yiwu, 322000 Zhejiang Province People’s Republic of China; 2 Rehabilitation Department, Yiwu Maternity and Child Health Care Hospital, Xinke Road C100, Yiwu, 322000 Zhejiang Province People’s Republic of China

**Keywords:** Bainbridge-Ropers syndrome, *ASXL3*, nonsense variant, intellectual disability

## Abstract

**Background:**

Bainbridge-Ropers syndrome (BRPS) [OMIM#615485] is a neurodevelopmental disorder, characterized by delayed psychomotor development with generalized hypotonia, moderate to severe intellectual disability, poor or absent speech, feeding difficulties, growth failure, dysmorphic craniofacial features and minor skeletal features. The aim of this study was to investigate the genetic etiology of a Sudanese boy with severe developmental delay, intellectual disability, and craniofacial phenotype using trio-based whole-exome sequencing. To our knowledge, no patients with *ASXL3* gene variant c.3043C>T have been reported detailedly in literature.

**Case presentation:**

The patient (male, 3 years 6 months) was the first born of a healthy non-consanguineous couple originating from Sudan, treated for “psychomotor retardation” for more than 8 months in Yiwu. The patient exhibited severely delayed milestones in physiological and intellectual developmental stages, language impairment, poor eye-contact, lack of subtle motions of fingers, fear of claustrophobic space, hypotonia, clinodactyly, autistic features. Peripheral blood samples were collected from the patient and his parents. Trio-based whole-exome sequencing(Trio-WES) identified a de novo heterozygous *ASXL3* gene variant c.3043C>T;p.Q1015X. Sanger sequencing verified variants of this family.

**Conclusion:**

Trio-WES analysis identified a de novo nonsense variant (c.3043C>T) of *ASXL3* gene in a Sudanese boy. To our knowledge, the patient with this variant has not been reported previously in literature. This study presents a new case for *ASXL3* gene variants, which expanded the mutational and phenotypic spectrum.

## Background

Bainbridge-Ropers syndrome (BRPS) is a severe neurodevelopmental disorder characterized by failure to thrive, severe developmental delay, intellectual disability, and craniofacial phenotype. BRPS is caused by a heterozygous loss-of-function mutation in *ASXL3* gene. With the use of trio-based whole-exome sequencing, we reported a Sudanese boy with a novel pathogenic variant in *ASXL3* gene. We described clinical abnormalities, medical imaging and genetic characteristics of this disease to improve the comprehensive understanding of BRPS for pediatricians.

## Case presentation

**Fig. 1:**
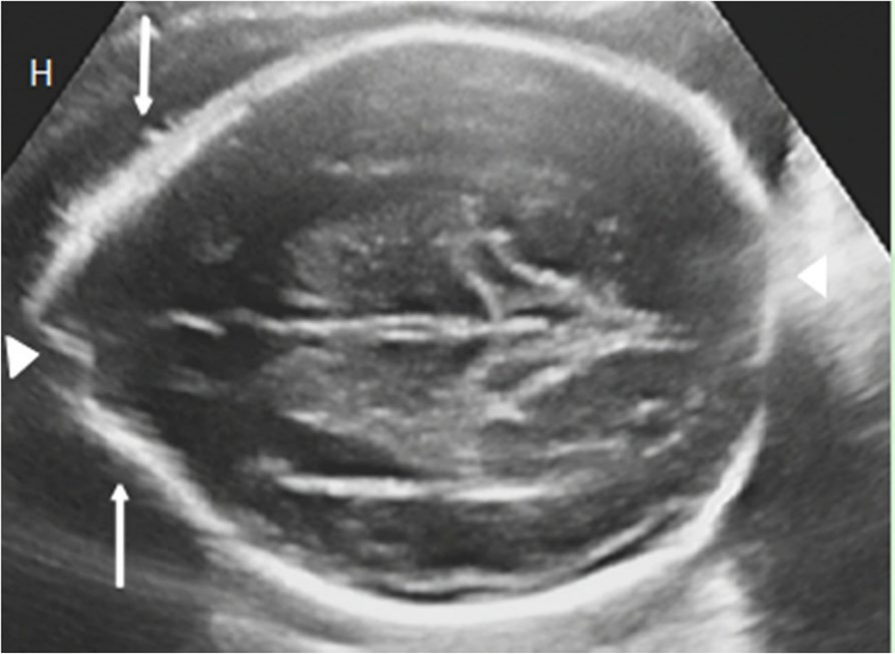
The 29th week of gestation, the fetus with scaphocephaly. Axial view of the fetal head (H) showed a long (arrowheads) and narrow head (arrows)

**Fig. 2:**
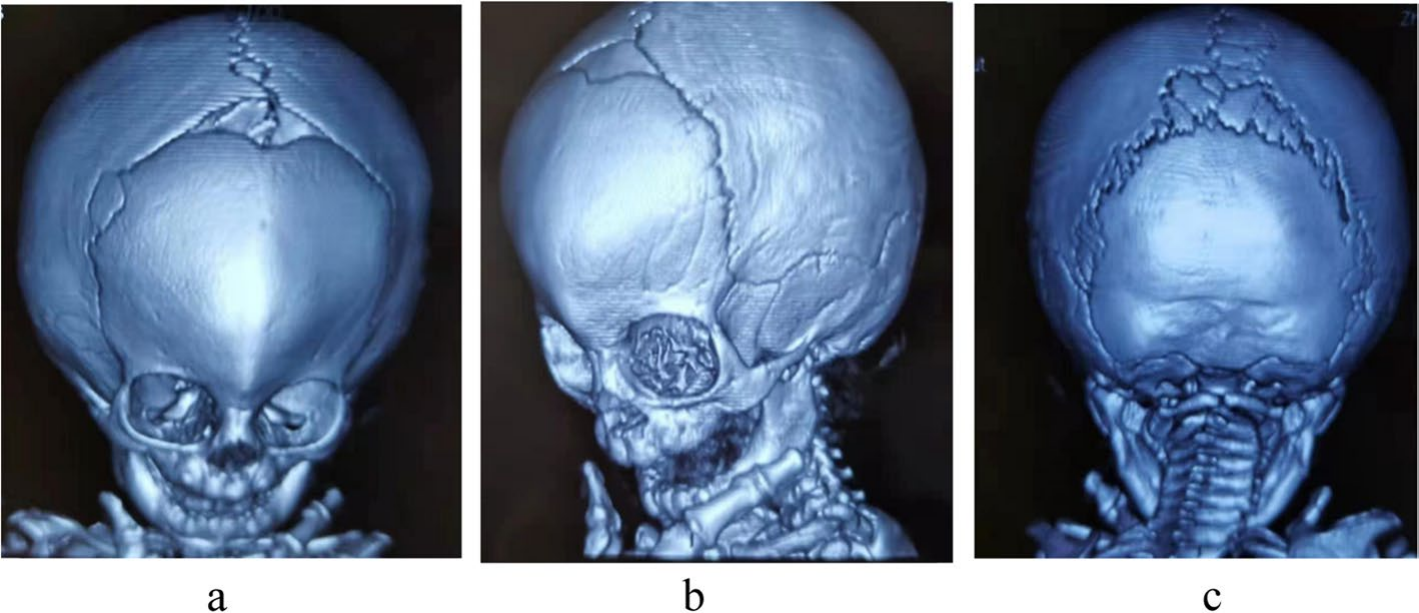
a-c at 11 months of age, three-dimensional CT imaging of the patient

The patient (male, 3 years 6 months) was born of a healthy non-consanguineous couple originating from Sudan, treated for “psychomotor retardation” for more than 8 months in Yiwu. Family history was unremarkable. At 37 weeks, the patient was born in the first child of his mother by cesarean delivery. The mother denied the history of birth asphyxia and intracranial hemorrhage. The mother’s pregnancy was abnormal with hypothyroidism and gestational diabetes mellitus. There was no exposure to alcohol, tobacco, or drugs. In the 29th week of gestation, ultrasonography indicated scaphocephaly (scaphocephaly occurs when there is a premature fusion of the sagittal suture) (Fig. 1). The birth weight was 2.66 kg and length was 32 cm. After breast-fed up for 3 months, the patient presented feeding difficulties. At 11 months of age, the patient could not lift his head. Three-dimensional CT imaging showed premature fusion of cranial suture, microcephaly and trigoncephaly (Fig. 2), then the patient received craniostenosis surgery (extensive cranioplasty for sagittal synostosis by preserving cranial bone flaps adhered to the dura mater) [1] in Hainan children’s hospital. One month after surgery, the patient was transferred to our hospital for rehabilitation. The results of action observation training (AOT) and goal-directed training (GDT) showed promising.

**Fig. 3:**
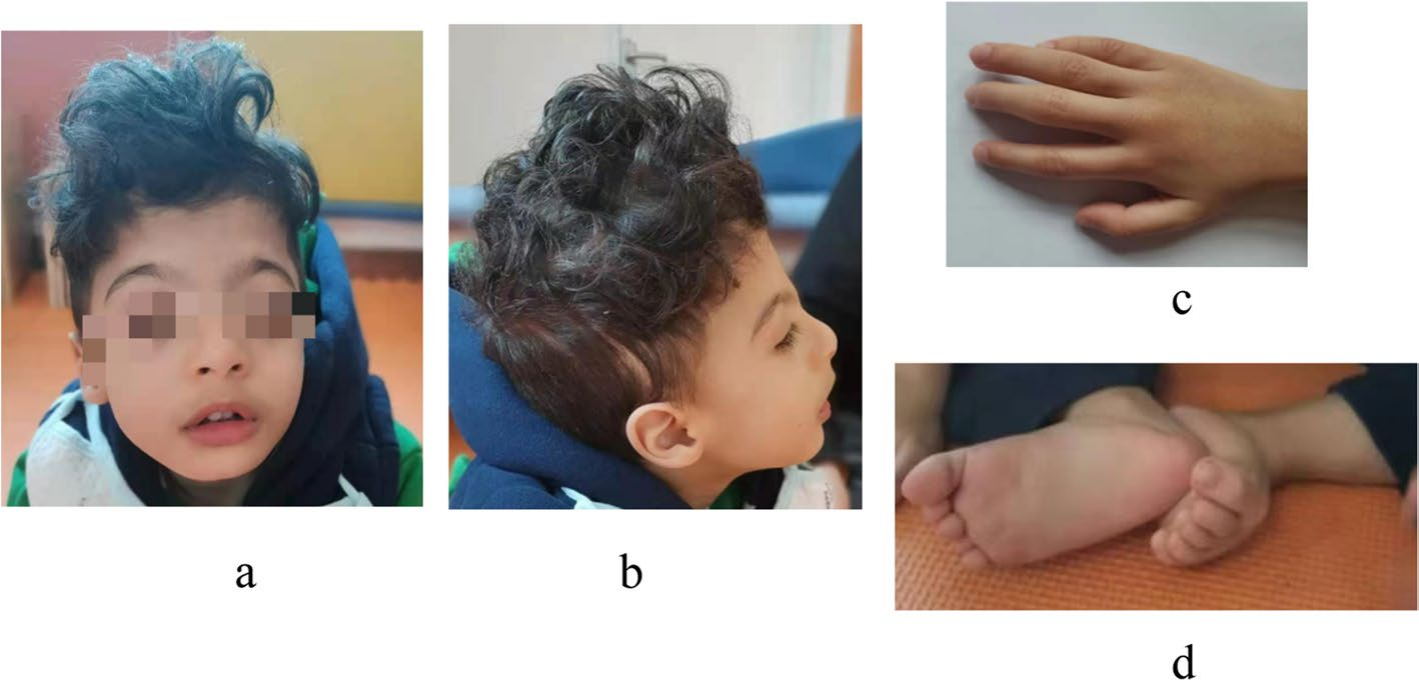
**a-b** microcephaly, strabismus, widely spaced eyes, epicanthal folds, highly arched eyebrows, long lashes, depressed nasal ridge, crowded teeth, thin upper lip vermillion, hirsutism, posteriorly rotated ear, anteverted nares, and palate malformations. **c** the hand were relaxed, not clawed. **d** the feet clenched like fists

**Fig. 4:**
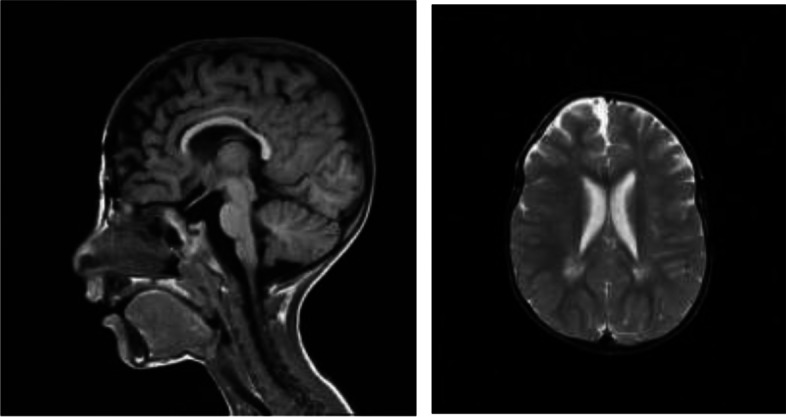
at 3 years 6 months of age, brain MRI of the patient indicated that the vascular space in the posterior horn of the ventricle was widened

At 3 years 6 months of age, the patient had a weight of 8.0 kg(< 3^rd^ percentile), a length of 50 cm and an occipital frontal circumference (OFC) of 43.8cm(< 3^rd^ percentile). The patient exhibited severely delayed milestones in physiological and intellectual developmental stages(IQ<50), language impairment, poor eye-contact, lack of subtle motions of fingers, fear of claustrophobic space, hypotonia, clinodactyly. He had autistic features as not playing with other children of his own age and a narrow range of interests. He had obvious self-harm behavior, manifested as head banging. At present, the patient could lift his head, roll over, but could not crawl or stand on four limbs. He was able to sit alone without support for only moments. He could not make a fist. In the supine position, he had active grasp consciousness, could grasp cubes using the ulnar-palmar grasp technique; but in the sitting prone position, he couldn’t do these. His eyes could not track objects, and he had no response to sound or simple verbal commands. He could not says simple words, such as "Mama" and "Dada". Bilateral patellar reflex was present, Babinski reflex was negative, step reflex was not induced. He has facial dimorphism (Fig. 3), including microcephaly, strabismus, widely spaced eyes, epicanthal folds, highly arched eyebrows, long lashes, depressed nasal ridge, crowded teeth, thin upper lip vermillion, hirsutism, posteriorly rotated ear, anteverted nares, and palate malformations. The brain interictal electroencephalogram (EEG) showed normal and magnetic resonance imaging (MRI) indicated that the vascular space in the posterior horn of the ventricle was widened (Fig. 4).

## Neuropsychological scores

The Griffiths mental development scales for China (GDS-C) are used to assess the development of children from birth to 8 years across six separate subscales: locomotor (A), personal-social (B), language (C), eye-hand co-ordination (D), performance (E) and practical reasoning (F). Developmental curves with respect to all the six subscales together with the “general quotient”(GQ) were plotted. The GQ was derived by calculating the average of the raw scores of the six subscales. The raw scores of the six subscales were converted to the corresponding percentiles. The 1st, 5th, 10th, 25th, 50th, 75th, 90th, 95th, and 99th percentiles(the percentile graphs demonstrated normative trends in developmental scores)were displayed in each plot [2]. The patient had a GQ of 16.25. (The raw scores of the five subscales were lower than 1st percentile, the score of subscale F was not finished due to the child could not cooperate)

The childhood autism rating scale (CARS) is widely used by psychiatrists for identifying children with autistic spectrum disorder (ASD). The CARS score of the patient was 45 [not autistic < 30, mild or moderately autistic (30-36.5) or severely autistic > 36.5] [3].

The screening tool for autism in toddlers and young children (STAT )[4] is a Level 2 screening measure for children between 24 and 36 months and consists of 12 activity-based items that assess a range of socialcommunicative behaviors. The patient obtained a STAT score of 3.0 (a score greater than or equal to the cutoff (1.75) indicates autism risk).

## Genetic tests and laboratory examination

Parents signed an informed consent for genetic analysis. Our legal ethics committee approved this genetic study. DNA isolated from peripheral blood cells was enriched for trio-WES and Sanger sequencing was used for verification. The patient was screened for genetic metabolic disease, chromosome karyotyping, and copy number variation (CNV). Levels of urine organic acids, plasma amino acids, blood gas analysis, lactate, pyruvic acid and thyroid function were normal. Conventional G-banded chromosome analysis showed a 46,XY karyotype.

**Fig. 5:**
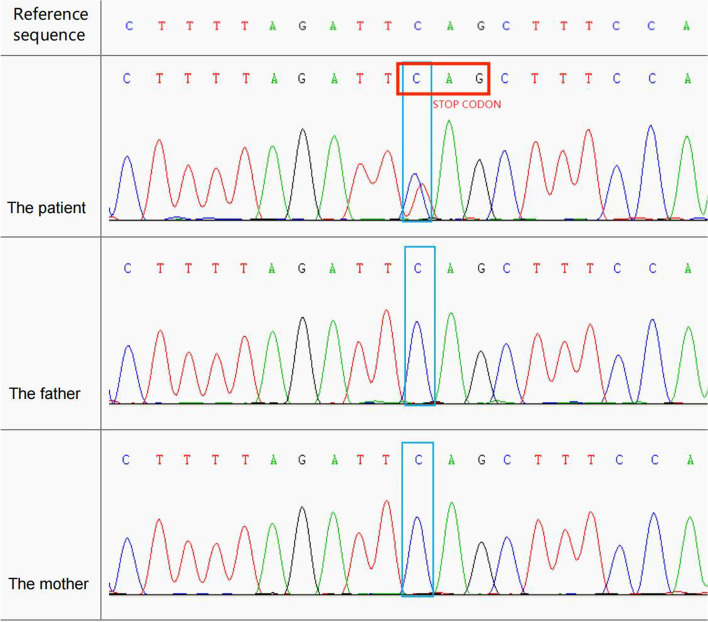
**a-c** Sanger sequencing of the patient and parents a, A de novo heterozygous *ASXL3* gene variant c.3043C>T;p.Q1015X(NM_030632.3) in the patient. **b**, Not found in his father. c, Not found in his mother. The frame indicates the location of the variant

The patient was examined using a multi-step molecular diagnostics algorithm, including genetic metabolic disease, chromosome karyotyping, and CNV with negative results. Trio-based whole-exome sequencing identified a de novo heterozygous *ASXL3* gene variant c.3043C>T;p.Q1015X(NM_030632.3)(Table 1). This variant has not been reported in the dbSNP, gnomAD, the 1,000 Genomes Project, Exome Aggregation Consortium and Exome Variant Server. The de novo status was independently confirmed by Sanger sequencing of parents (Fig. 5).

**Table 1:** Genomic findings and variant interpretation

Gene	Genomic location	HGVS cDNA	HGVS protein	Zygosity	Parent of origin	Interpretation
*ASXL3*	Chr18:31322918 C>T(GRCh37)	c.3043C>T (NM_030632.3)	p.Q1015X	Het	De novo	Pathogenic (PVS1,PS2, PM2,PP4,PP5)

Criteria: PVS1: null variant; PS2: de novo in a patient with disease and no family history; PM2: absent from controls; PP4: patient’s phenotype is highly specific for a disease with a single genetic etiology; PP5: reputable source recently reports the variant as likely pathogenic, but evidence was not available for us to perform an independent evaluation.

## Discussion and conclusions

Bainbridge-Ropers syndrome (BRPS) is inherited in an autosomal dominant model and was first described in 2013. Bainbridge et al [5] reported de novo truncating mutations in *ASXL3* in four unrelated probands with psychomotor retardation, failure to thrive, feeding difficulties, language delay and dysmorphic craniofacial features.To date, more than 30 cases were described in literature [6]. The clinical phenotype of the Sudanese boy has not showed morbid obesity [2], generalized epilepsy [7], undescended testes[8], nystagmus [9], pontocerebellar hypoplasi a[10], hypoplasia of corpus callosum [11], nystagmus [12], which described previously in Bainbridge-Ropers syndrome. Mutations in *ASXL3* may also cause congenital hypothyroidism [13] and autosomal recessive congenital heart disease [14].

The *ASXL transcriptional regulator 3* (*ASXL3*) gene is located at the 18q12.1 chromosome region and encodes putative polycomb group protein ASXL3. ASXL3 contains a plant homeodomain (PHD) zinc finger domain, which is a member of the vertebrate ASX-like protein family playing a role in the regulation of gene transcription. Protein ASXL3 expressed mainly in brain, testis and ovary [15]. This transcript of *ASXL3*(NM_030632.3) has 12 coding exons, transcript length of 11,760 bps, and translation length of 2,248 residues.

To our knowledge, no patients with this variant have been reported detailedly in literature. We interpret c.3043C>T(p.Q1015X) as a pathogenic variant. The variant causes a premature termination codon, resulting in the strong protein truncation (more than 50% of protein length is missing).

For now, the number of variants reported in literature and our study is 37. Except for one splicing variant, all of them are nonsense and frameshift variants, which are mostly located in two mutational cluster regions (MCRs) distributed in the 5’ end of exon 11 and exon 12 respectively [16]. These variants reported thus far are predicted to result in protein truncation and are prone to nonsense mediated decay, with the resultant reduction in the expression of *ASXL3*. Thus haploinsufficiency may represent the mechanism of Bainbridge-Ropers syndrome.

In the previous literature, no patients with BRPS received craniostenosis surgery. There are no reports of BRPS in Sudanese with de novo truncating mutations. This study expands the clinical phenotype of BRPS and *ASXL3* gene mutational spectrum.

In summary, we identified a novel heterozygous *ASXL3* gene variant c.3043C>T(p.Q1015X) in a Sudanese family, which had not been reported previously. The clinical phenotype like feeding difficulties, intellectual disability, failure to thrive, and craniofacial features in children are always difficult to distinguish. Whole-exome sequencing is a useful tool for precise diagnosis, carrier screening and genetic counselling. The skull of young children is relatively thin and early surgery can easily achieve satisfactory bone reshaping avoiding skull deformity. Such surgical technique could be considered for these patients if necessary.

## Data Availability

The datasets used and/or analyzed during the current study are available from the corresponding author on reasonable request.

## Abbreviations

BRPS: Bainbridge-Ropers syndrome
ASXL3: Additional sex combs-like 3
Trio-WES: Trio-based whole-exome sequencing
MCR: Mutational cluster regions
CNV: Copy number variation
CT: Computed Tomography
AOT: Action observation training
GDT: Goal-directed training
MRI: Magnetic resonance imaging
GQ: General quotient
CARS: Childhood autism rating scale
GDS-C: Griffiths mental development scales for China
STAT: Screening tool for autism in toddlers and young children
